# *Synsepalum chimanimani* (Sapotaceae), a new species from the Chimanimani Mountains of Mozambique and Zimbabwe, with notes on the botanical importance of this area

**DOI:** 10.3897/phytokeys.133.38694

**Published:** 2019-10-16

**Authors:** Saba Rokni, Bart Wursten, Iain Darbyshire

**Affiliations:** 1 Herbarium, Royal Botanic Gardens, Kew, Richmond, TW9 3AE, UK Herbarium, Royal Botanic Gardens Richmond United Kingdom; 2 Herbarium, Nieuwelaan 38, Meise 1860, Belgium Herbarium Meise Belgium

**Keywords:** conservation, herbarium, Important Plant Area, Makurupini, taxonomy

## Abstract

*Synsepalum chimanimani* S.Rokni & I.Darbysh., **sp. nov.**, a small tree endemic to the forests of the southern foothills of the Chimanimani Mountains of Mozambique and Zimbabwe, is described and illustrated. The differences in morphology and distribution between the new species and the related *S. kaessneri* and *S. muelleri*, with which it has been confused, are clarified. The new species is globally Endangered due to ongoing habitat loss within its restricted range. The botanical importance and conservation of the Chimanimani foothills is also discussed, and they are highlighted as a candidate Important Plant Area.

## Introduction

*Synsepalum* (A.DC.) Daniell is a genus of approximately 35 species of evergreen shrubs or trees native to tropical Africa (Pennington 1991; Borg et al. 2019). The species and groups of species in this genus are often very distinct, which has caused previous authors to attempt to subdivide it into a number of smaller genera including *Afrosersalisia* A.Chev., *Pachystela* Radlk. and *Vincentella* Pierre (see, for example, Hemsley 1968; Kupicha 1983), resulting in an extensive synonymy. However, the characters used to define these genera have a reticulate pattern of variation, and the lack of correlation between these characters precludes any subdivision of this group except on a single character basis, which results in different groupings according to which character is weighted. This unsatisfactory generic classification led to the segregate genera being united under *Synsepalum* by Pennington (1991).

In a recent molecular phylogenetic study by Borg et al. (2019), which used nuclear ribosomal DNA and plastid trnH–psbA sequences to elucidate relationships within and between *Synsepalum* and *Englerophytum* K.Krause, neither genus was supported as monophyletic. This study identified six major lineages within this clade, and provided morphological evidence in support of these lineages. However, only 11 species of *Synsepalum* and eight (out of 19) species of *Englerophytum* were sampled in this study, and the authors concluded that more morphological and molecular data are required before making final taxonomic decisions regarding the placement of species in this clade and whether or not to reinstate some of the former segregate genera.

The new species of *Synsepalum* described here, recorded from the lowland forests of the Chimanimani Mountains on the border of Mozambique and Zimbabwe, has previously been treated as conspecific with *S. kaessneri* (Engl.) T.D.Penn. (Kupicha 1983, as *Afrosersalisia kaessneri* (Engl.) J.H.Hemsl.). *Synsepalum kaessneri* is otherwise a rare species of lowland forest in the Eastern Arc Mountains and adjacent lowlands of Kenya and Tanzania, with the nearest populations nearly 1,500 km NNE of the Chimanimani Mountains. However, few previous collections were available from Mozambique and Zimbabwe, and only fruiting and sterile specimens had been collected until recently. Further specimens of the Chimanimani *Synsepalum* species were collected in October 2013 in the foothills of the southern Chimanimani massif in Mozambique by Bart Wursten, including the first specimen from this area bearing flowers (*Wursten BW897*). Additional specimens of the same taxon from the same area were collected by M. Cheek and J. Timberlake et al. in 2015 under a Darwin Initiative project focussing on the conservation of the Chimanimani forest zone in Mozambique (Timberlake et al. 2016a). Examination and comparison of these new collections with existing collections of *Synsepalum kaessneri* showed that, although the plants collected in Mozambique and adjacent Zimbabwe are very similar and closely related to *Synsepalum kaessneri*, they differ in several characters and so are considered to be different, allopatric species.

In their comprehensive guide to the trees and shrubs of Mozambique, Burrows et al. (2018, p. 744) included the Chimanimani taxon within *Synsepalum muelleri* (Kupicha) T.D.Penn. (formerly *Vincentella muelleri* Kupicha), a species that occurs in submontane forest in northern Mozambique and southern Malawi (Kupicha 1983). The plants in the photographs in Burrows et al. (2018) (*Wursten BW887* and *BW897*) are the Chimanimani taxon rather than *Synsepalum muelleri* sensu stricto. There are several notable differences between the two taxa, and they are clearly not conspecific.

In this paper we describe the Chimanimani taxon as a new species and clarify the differences in morphology and distribution between our new species and *S. kaessneri* and *S. muelleri*. We also present a distribution map and list of specimens examined for the three species and discuss the conservation and botanical importance of the Chimanimani foothills.

## Methods

Specimens of *Synsepalum* species in the Kew herbarium (**K**), the British Museum of Natural History (**BM**), Institute for Agricultural Research of Mozambique (**LMA**), National Herbarium and Botanic Garden, Harare (**SRGH**) and on loan from Meise Botanic Garden Herbarium (**BR**) were examined. Online herbaria **BR** (http://www.botanicalcollections.be/#/en/home), **LISC** (http://maerua.iict.pt/colecoes/herb_simplesearch.php), **WAG** (https://bioportal.naturalis.nl/?language=en), **E** (https://data.rbge.org.uk/search/herbarium/), **MO** (https://www.tropicos.org/) and **US** (https://collections.nmnh.si.edu/search/botany/) (acronyms according to Thiers 2019), JSTOR Global Plants and the Flora of Mozambique website (Hyde et al. 2019) were also checked and all specimens with images available are cited. The countries in the “Specimens examined” sections are listed alphabetically.

Thirty qualitative and quantitative characters were examined in specimens of *S. kaessneri* and the newly described species (see Suppl. material 1). Measurements were made using a Leica Wild M8 stereo microscope and a graduated ruler (0.5 mm graduations). All characters were measured on dry material, except internal floral characters which were measured from a rehydrated flower. The terminology used follows Beentje (2010). The distribution map (Fig. 3) was produced in SimpleMappr (https://www.simplemappr.net) using georeferenced point localities.

In the Discussion section the application of Important Plant Areas (IPA) criteria follows Darbyshire et al. (2017). The abbreviations used for IUCN Red List assessments follow IUCN (2012).

## Taxonomic treatment

### Synsepalum chimanimani

Taxon classificationPlantaeEricalesSapotaceae

S.Rokni & I.Darbysh. sp. nov.

0024D73A-3A44-5014-B99E-8CA7FAEF8237

urn:lsid:ipni.org:names:77202384-1

Figures 1A–K , 2

Tulestea kaessneri sensu Aubréville in Adansonia Sér. 2, 12(2): 191–192 (1972), pro parte quoad *Wild*, *Goldsmith & Müller 6645*, non (Engl.) Aubrév. sensu stricto.
Afrosersalisia kaessneri sensu Kupicha in Flora Zambesiaca 7(1): 217 (1983), pro parte quoad spec. ex Mozambique & Zimbabwe, non (Engl.) J.H.Hemsl.
Synsepalum kaessneri sensu Pennington in Gen. Sapotac.: 249 (1991), pro parte, non (Engl.) T.D.Penn. sensu stricto.
Synsepalum muelleri sensu Burrows et al. in Trees & Shrubs Mozambique: 744 (2018), pro parte – photographs and reference to distribution in Haroni-Makurupini Forest; non (Kupicha) T.D.Penn.
Synsepalum sp. aff. S. kaessneri (Engl.) T.D.Penn., Hyde et al. in Flora of Mozambique (2019).

#### Type.

MOZAMBIQUE. Manica Province: Magorogodo hills, Zomba Community, 19°54'28"S, 33°11'4"E, c. 559 m alt., fl. and fr. 28 October 2013, *B.T. Wursten BW897* (holotype: BR!, BR0000020700003)].

#### Diagnosis.

**Figure 1.**
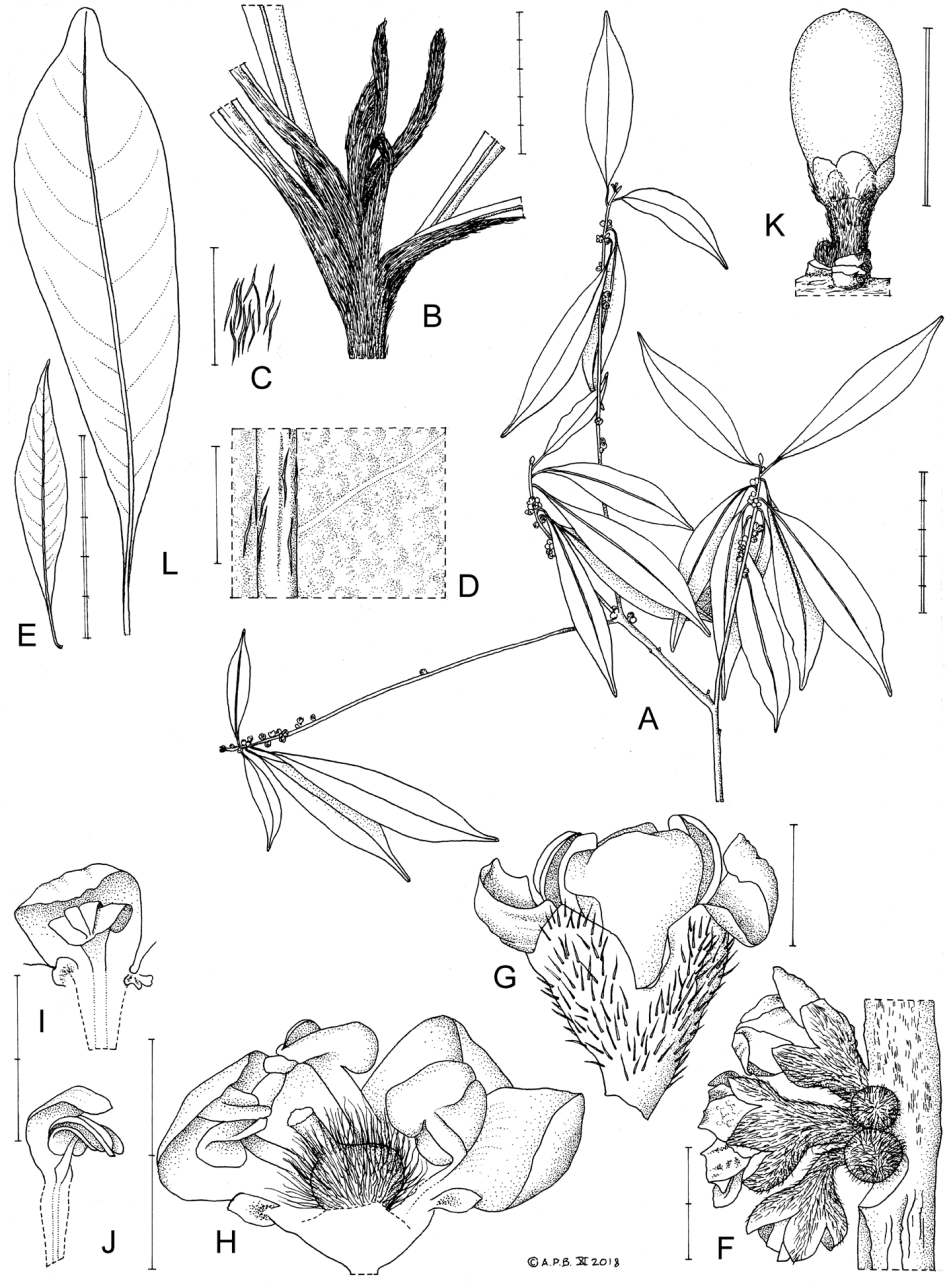
*Synsepalum chimanimani* (**A-K**) and *Synsepalum kaessneri* (L) **A** habit **B** stem apex with apical buds/young leaves and petioles showing indumentum **C** medifixed hairs on stem **D** abaxial leaf surface showing sparse medifixed hairs on midrib **E** leaf showing (faint) secondary veins **F** flower cluster showing bud, open flower and partially opened flower **G** flower, side view (hydrated) **H** corolla after removal of two petals and stamens (hydrated) **I** stamen and staminodes in situ on petal, inner face bases of neighbouring petals shown **J** side view of stamen and petal (staminodes omitted) **K** immature fruit (from photograph) **L** leaf (abaxial) of *Synsepalum kaessneri.***A, D-K** drawn from *B.T. Wursten BW897* (BR0000020700003) **B, C** from *Timberlake et al. 6197* (K001291035) **L** drawn from *Magogo & Glover 280* (K). Scale bars: 1mm (Single bar); 2 mm and 5 mm (graduated single bar); 1 cm (double bar); 5 cm (graduated double bar). Drawn by Andrew Brown, November 2018.

This species differs from *Synsepalum kaessneri* (Engl.) T.D.Penn. in the generally smaller (7.9–12.6 x 1.7–3.4 cm versus 9.8–16.7 x 2.8–5.2 cm) narrowly elliptic leaves with a long and narrow acuminate tip versus oblanceolate leaves with a short and broad acuminate tip (see illustration, Fig. 1E, L); flowers sessile or almost so with pedicels less than 1 mm long (extending to 2 mm long in fruit) versus flowers stalked with pedicels 1–3 mm long (extending to 3–5 mm in fruit); shorter corolla tube (0.75–0.8 mm long versus 1.2 mm long) and shorter (1.45–1.5 mm versus 1.8–1.9 mm), broadly ovate versus ovate corolla lobes; anthers with elliptic thecae with a minute, inconspicuous point at the apex of the connective versus arrow-head shaped anthers with oblong thecae with a conspicuous apiculate apex to the connective. Table 1 shows the distinguishing characters between the two species.

**Table 1.** Diagnostic characters for separating *Synsepalum chimanimani* from *Synsepalum kaessneri*.

Character	*Synsepalum chimanimani*	*Synsepalum kaessneri*
Leaf shape	Narrowly elliptic to rarely oblanceolate	Oblanceolate
Leaf apex	long acuminate, tip narrow, rounded	short acuminate with a broad rounded tip
Leaf width (mm)	17–34	28–52
Leaf length (mm)	79–126	98–167
Leaf length: width ratio	3.1–5.83	2.7–4.14
Pedicel length (mm) – flowers	Flowers sessile or almost so – pedicel less than 1 mm long	Flowers stalked – pedicel 1–3 mm long
Pedicel length (mm) – fruit	2	3–5
Corolla lobes – shape	Broadly ovate	Ovate
Corolla lobes length (mm)	1.45–1.5	1.8–1.9
Corolla lobes length: width ratio	1–1.21	1.29–1.36
Corolla tube length (mm)	0.75–0.8	1.2
Corolla – total length (mm)	Less than 2.5 mm	c. 3 mm
Stamens	Anthers 0.9–1 mm long, thecae elliptic with a minute, inconspicuous point at the apex of the connective	Anthers 1.25 mm long, arrow-head shaped, thecae oblong with conspicuous apiculate apex to connective

**Figure 2.**
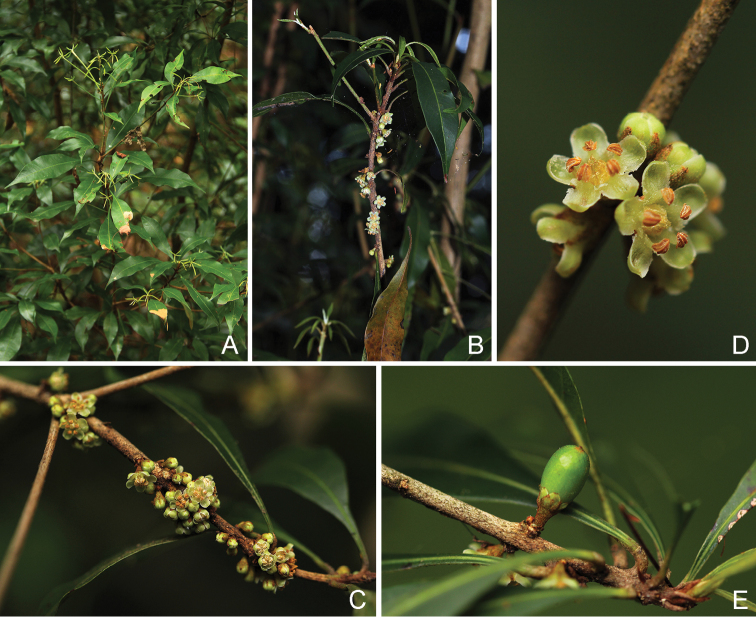
*Synsepalum chimanimani***A** habit and leaves **B, C** flowering stems **D** flowers **E** immature fruit (Photographs by Bart Wursten).

It has previously been confused with *Synsepalum muelleri* (Kupicha) T.D.Penn. but is easily separated by the very faint secondary venation and no visible tertiary venation versus clearly visible secondary and reticulate tertiary venation; no stipules versus persistent subulate stipules 2–7 mm long; whitish-green flowers with a very short corolla tube and wide-spreading lobes versus white tubular flowers with the tube markedly longer than the lobes; corollas less than 2.5 mm long versus 7–10 mm long; exerted stamens versus included stamens; minute staminodes versus no staminodes; fruit 1–1.3 cm long with a very short (0.5 mm long) style versus larger fruit 2–3 cm long with a 5–7 mm long persistent style; glabrous fruit except for hairs at the tip around the base of the style versus fruit covered with rust-coloured hairs.

#### Description.

Small tree or shrub up to 4 m high. Bark brown and finely fissured. Branching repeatedly subterminal (“*Terminalia*-style”) with leaves confined to branch apices. Older stems glabrous with finely fissured brownish-grey bark, young shoots, buds and petioles of young leaves with indumentum of appressed, very fine rust-coloured hairs. Hairs medifixed, less than 0.5 mm long, tips sharp. Stipules absent. Leaves with petiole 3–5 mm long, often sparsely hairy to glabrous on older leaves; lamina narrowly elliptic to rarely oblanceolate, 7.9–12.6 x 1.7–3.4 cm, apex long acuminate with a narrow rounded tip, margins entire and repand, base attenuate; midrib raised on both surfaces with striations on the midrib below, lateral veins very faint above, more distinct below, pinnate, curving towards the margin, 9–15 pairs of lateral veins, no visible tertiary venation; both surfaces finely rugose and glabrous except for sparse scattered hairs along the midrib on the lower surface, particularly on younger leaves; hairs very fine, rust-coloured, medifixed, less than 0.5 mm long, tips sharp. Flowers in clusters in leaf axils and along the branches below the leaves, small and whitish-green, sessile or almost so, pedicels less than 1 mm long (extending to 2 mm long in fruit), pedicels and external surface of calyx lobes covered with sub-appressed, medifixed, very fine rust-coloured hairs. Calyx cup-shaped, 5-lobed, lobes nearly free, imbricate, elliptic, 1.2–1.3 x 1–1.1 mm, with hyaline margins, glabrous internally. Corolla 5-merous, fused at base, tube up to 0.8 mm long, lobes involute, broadly ovate, 1.45–1.5 mm long, rounded at apex. Stamens attached at base of petals, filaments 0.6 mm long, anthers 0.9–1 mm long, the two thecae elliptic with a tiny, inconspicuous point at the apex of the connective. Staminodes minute, alternating with the petals and stamens, divided and irregularly shaped. Ovary densely hairy, with a few hairs extending onto the style, ovoid, 1mm long, style 0.5 mm long. Fruit fleshy, red when ripe, solitary, ellipsoid, 10–13 x 6–8 mm (measurements taken from dried material), with calyx and corolla persisting at base and with persistent style at apex, glabrous except for a few hairs at apex around base of style. Seed compressed-ellipsoid, 12 x 7 x 3 mm, glossy brown, with duller elliptic scar c. 5 mm wide extending the length of the seed, cotyledons large, plano-convex, endosperm absent (fide Kupicha 1983).

#### Distribution and ecology.

Known only from lowland, moist forests in the foothills of the southern Chimanimani mountains of Mozambique and Zimbabwe. It has been recorded from three localities: the Haroni-Makurupini Forest in Zimbabwe, and the Maronga forest (Maronga Community) and Thekeza forest (Zomba Community) in Mozambique. It occurs in the understorey of moist evergreen and semi-deciduous forests at an altitude of 305–560 m. In the Maronga Community area at the base of the Chimanimani Mountains, where this species is locally frequent, most of the area is covered by moist evergreen forest. The dominant tree here is *Newtonia buchananii*(Baker f.) G.C.C.Gilbert & Boutique with *Maranthes goetzeniana* (Engl.) Prance and *Xylopia aethiopica* (Dunal) A.Rich. also common. *Funtumia africana* (Benth.) Stapf forms a high sub-canopy along with *Aporrhiza nitida* Gilg, *Blighia unijugata* Baker, *Millettia stuhlmannii* Taub., *Synsepalum brevipes* (Baker) T.D.Penn. and *Trilepisium madagascariense* DC. The understorey is dominated in some areas by *Drypetes arguta* (Müll. Arg.) Hutch. and other locally abundant shrubs include *Rinorea convallarioides* (Baker f.) Eyles, *Rinorea ferruginea* Engl., *Tabernaemontana ventricosa* Hochst. ex A.DC., *Tricalysia pallens* Hiern and the rare *Vepris drummondii* Mendonça. There are also many lianas (Timberlake et al. 2016a).

#### Phenology.

Plants were collected in flower in July (buds) and October (open), and in fruit in October and December. Flowering occurs at the end of the dry season and beginning of the rainy season, the main rainy season in the Chimanimani area being from November to late March or April (Timberlake et al. 2016b).

#### Etymology.

The specific epithet is taken from the Chimanimani mountains to which the species is confined.

#### Conservation status.

*Synsepalum chimanimani* S.Rokni & I.Darbysh, sp. nov. has been assessed as Endangered under IUCN criterion B (EN B1ab(iii)+2ab(iii); Rokni et al. 2018, as *Synsepalum* sp. nov.). It is estimated to have an extent of occurrence (EOO) and area of occupancy (AOO) of only 16 km^2^ and is known from fewer than five locations. Although part of its population is well protected within the core zone of the Chimanimani Trans-Frontier Conservation Area (TFCA), it is threatened by extensive destruction and degradation of its forest habitat within the buffer zone of the TFCA, particularly within the Maronga and Zomba communities of Mozambique (see Discussion for further information on threats in this area).

#### Specimens examined.

**MOZAMBIQUE.** Manica Province: Magorogodo hills, Zomba Community, 19°54.467'S, 33°11.067'E, alt. c. 559 m, fl. and fr. 28 October 2013, *Wursten BW897* (BR!, BR0000020700003); Survey Plot 3, Magorogodo hills, Zomba Community, alt. 548 m, st. 28 October 2013, *Wursten BW887* (BR!); Sussundenga Dist., Maronga community, base of Chimanimani Mountains, Forest plot 002, 19°58.417'S, 33°5.233'E, alt. 341 m, st. 14 November 2015, *Timberlake et al. 6196* (LMA!); Sussundenga Dist., Maronga community at base of Chimanimani Mountains, 19°58.928'S, 33°4.948'E, alt. 330 m, st. 17 November 2015, *Timberlake et al. 6197* (K!, K001291034; LMA!); Sussundenga Dist., Chimanimani foothills, Zomba community, Thekeza Forest, 19°54.717'S, 33°11.433'E, alt. 386 m, st. 30 June 2015, *Cheek 17963* (K!, K001291036); Sussundenga Dist., Chimanimani foothills, Zomba community, Last House Thekeza, 19°54.533'S, 33°11.303'E, alt. 542 m, fl. buds 2 July 2015, *Cheek 18027* (K!, K001291037); Southern tip Chimanimani Mts. near Haroni-Makurupini Forest, alt. +/- 1000 ft [c. 305 m], st. 28 May 1969, *Müller 1085* (SRGH!). **ZIMBABWE.** Manicaland Province: Chimanimani District, Haroni/Makurupini Forest, alt. 1300 ft [396 m], fr. 4 December 1964, *Wild*, *Goldsmith & Müller 6645* (K!, K001291026; BR!, BR0000020184520; SRGH!); Chimanimani District, Makurupini-Haroni Forest, st. 22 April 1973, *Mavi 1437* (K!, K001291028; SRGH!).

**Figure 3.**
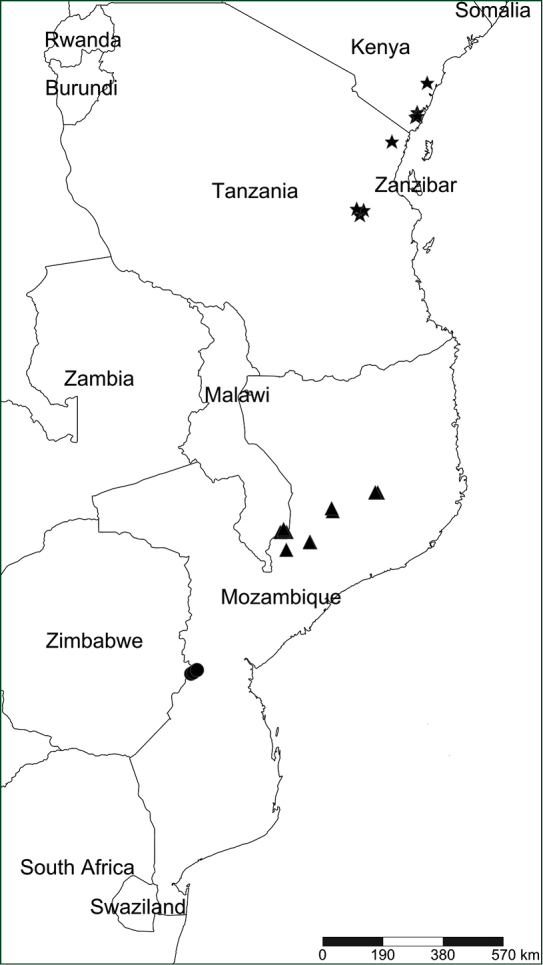
Geographical distribution map of *S. chimanimani*, *S. kaessneri*, and *S. muelleri*. Circles – *S. chimanimani*; triangles – *S. muelleri*; stars – *S. kaessneri*.

### Synsepalum kaessneri

Taxon classificationPlantaeEricalesSapotaceae

(Engl.) T.D.Penn., Pennington in Gen. Sapotac.: 249 (1991)

E7989A10-7CC9-5AEE-B67E-78C991B34952

Sersalisia kaessneri Engl., Engler in Mon. Afr. Pflanzen. 8: 31 (1904) (genus queried by original author).
Pouteria kaessneri (Engl.) Baehni, in Candollea 9: 280 (1942) (genus queried by author).
Afrosersalisia kaessneri (Engl.) J.H.Hemsl., Hemsley in Kew Bull. 20: 483 (1966); in F.T.E.A., Sapotaceae: 44 (1968).
Tulestea kaessneri (Engl.) Aubrév., Aubréville in Adansonia Ser. 2, 12(2): 191–192 (1972), pro parte quoad *T. Kässner 398*.

#### Type.

KENYA. Kwale County: Makoni near Mombasa, fl. 20 March 1902, *T. Kässner 398* (B, holotype, destroyed; BM!, isotype, BM000925429; K!, isotype, K000430647).

#### Specimens examined.

**KENYA.** Kwale County: Makoni near Mombasa, fl. 20 March 1902, *Kässner 398* (B, holotype, destroyed; BM!, isotype, BM000925429; K!, isotype, K000430647); Shimba Hills, Makadara Forest, Kwale Area, alt. 320 m [1050 ft], fr. 6 May 1968, *Magogo & Glover 1017* (K!); Shimba Hills, Shimba Forest area near Kwale, alt. 381 m [1250 ft], fl. 15 March 1968, *Magogo & Glover 280* (K!; BR!, BR0000020184513). Kilifi County: Mangea, Top forest, 3°16.000'S, 39°43.000'E, alt. 500 m, fr. 9 Jan 1992, *Robertson 6563* (K!; US!, 02704855; MO, EA). **TANZANIA.** Tanga Region: East Usambara Mountains, Kwamgumi Plot 4, 4°56.000'S, 38°43.000'E, alt. 300 m, st. 23 July 2001, *Luke et al. 7510* (K!, EA). Morogoro Region: Kimboza Forest Reserve c. 48–50 km Morogoro to Matombo Road, alt. c. 300–350 m, st. 9–17 July 1983, *Rodgers*, *Hall and Mwasumbi WAR 2609* (K!, DSM); Mkungwe Catchment Forest Reserve, slopes NW of the ridge, alt. 775 m, fr. 25 Jan 2001, *Jannerup & Mhoro 0287* (K!, C); Uluguru Mountains, in the valley leading South from Bigwa Mission, alt. 700–1100 m, fl. 27–29 September 1988, *Pócs & Knox 88189/B* (K!)

Aubréville (1972) also cites a specimen from Gabon collected in December 1971: ‘*A. Hladik 1878*, Ile de l’éléphant, Makokou, petit arbre, forêt partiellement inondable. Fruit rouge vermillon mangé par les chimpanzés.’ No herbarium is cited, and we have not seen this specimen. From the illustration and description, this specimen seems to have fruit with a definite point to the apex and a persistent style longer than 1 mm, and longer petioles (1.5–2.5 cm versus less than 1 cm long) and the flowers are also slightly larger than *S. kaessneri* at 3.75 mm in total length. This is very likely to represent a different species and is excluded here. *S. kaessneri* is not listed in the recent Gabon checklist (Sosef et al. 2006).

#### Habitat and ecology.

Moist evergreen and dry semi-deciduous forests. Altitude 300–1100 m.

#### Phenology.

Flowering September & March. Fruiting January & May.

### Synsepalum muelleri

Taxon classificationPlantaeEricalesSapotaceae

(Kupicha) T.D.Penn., Pennington in Gen. Sapotac.: 249 (1991)

093F347D-DD12-5C27-B551-9F012D1C0C1D

Vincentella muelleri Kupicha in Candollea 33 (1): 37, fig. 4 (1978); Kupicha in Sapotaceae Flora Zambesiaca 7(1): 228 (1983).

#### Type.

MALAWI. Southern Region: Mulanje District, Ruo Gorge, alt. 900 m, fl. and fr. 1 September 1970, *T. Müller 1463* (K!, holotype, K000049396; SRGH, isotype).

#### Specimens examined.

**MALAWI.** Southern Region: Mulanje Mountains, Ruo Gorge at the Savani stream crossing, alt. 1250 m, fr. 30 September 1986, *Chapman & Chapman 8104* (K!, K000049398; E!, E00330780; MO); Mulanje Mountains, Litchenya Plateau, along the perimeter fire trace near the crater lip, alt. 1920 m, fr. 12 November 1986, *Chapman & Chapman 8209* (K!, K000049399; E!, E00330776; MO); Mulanje Mountains, Chisongeli Forest, Muluzi catchment, alt. 1650 m, fl. 30 September 1988, *Chapman & Chapman 9339* (K!, K000049397; E!, E00330775; MO); Mulanje, Ruo Gorge, fl. and fr. 26 August 1983, *Dowsett-Lemaire 942* (K!, K000049400; BR!, BR0000019420677); Mulanje District, Ruo Gorge, alt. 900 m, fl. and fr. 1 September 1970, *Müller 1463* (K!, holotype, K000049396; SRGH, isotype; P); Mulanje Plateau, st. 24 September 1929, *Burtt Davy 22131* (BR!, BR0000019420684; FHO). **MOZAMBIQUE.** Nampula Province: Ribáuè, Serra de Mepáluè, alt. c. 1600 m, fr. 9 December 1967, *Torre & Correia 16403* (LISC!, paratype, LISC002849; COI; WAG!, paratype, WAG.1530185); Ribáuè, serra de Ribáuè (Mepáluè), alt. c. 1500 m, fr. 28 January 1964, *Torre & Paiva 10301* (K!, K001236782; LISC!, paratype, LISC002850; LMU). Zambézia Province: Encosta da serra do Gúruè, via fábrica Junqueiro a Oeste dos Picos Namuli, confluência dos rios Malema e Cocossi, alt. c. 1650 m, fl. and fr. (immature) 6 November 1967, *Torre & Correia 15921* (LISC!, paratype, LISC002848); Mt Namuli, Uivelo area, Manho forest, E Namuli, 15°24.683'S, 37°2.267'E, alt. 1590 m, st. 23 November 2007, *Timberlake et al. 5283* (K!, K000614012); Mount Chiperone, 16°30.283'S, 35°43.333'E, alt. 1155 m, fr. 29 November 2006, *Harris et al. 69* (K!, K000545061); Mount Chiperone, 28 November 2006, *Patel HP7152* (K!, K000545123); Mount Mabu, 16°17.017'S, 36°23.567'E, alt. 1252 m, fl. and fr. 16 October 2008, *Mphamba et al. 50* (K!, K000614270); Mt Mabu, Mabu forest on SE side, 16°17.067'S, 36°23.483'E, alt. 1260 m, st. 15 October 2008, *Timberlake et al. 5402* (K!, K000614394); Mt Mabu, 16°17.167'S, 36°24.083'E, alt. 960 m, st. 26 October 2008, *Timberlake et al. 5461* (K!, K000614442) [cf. muelleri]; Mt Mabu, veg plot 012, 16°17.500'S, 36°23.550'E, alt. 1389 m, st. 20 October 2008, *Timberlake s.n.* (K!, K000614472); Mt Mabu, veg plot 022, 16°17.033'S, 36°23.100'E, alt. 1320 m, st. 22 October 2008, *Timberlake s.n.* (K!, K000614490).

#### Habitat and ecology.

In the understorey of *Newtonia buchananii*-dominated moist forest, *Garcinia kingaensis* Engl. dominated moist forest and dense humid riverine forest at elevations of 900–1920 m.

#### Phenology.

Flowering August to November. Fruiting August to January. There is a distinct rainy season in Mabu, Namuli, Ribáuè, Chiperone and Mulanje with the main rainfall months being November to April with a dry season from May to October. Flowering occurs at the end of the dry season and beginning of the rainy season with fruiting following.

## Discussion

### Taxonomy and conservation

We have only one flowering specimen of *S. chimanimani* sp. nov. and it has rarely been collected in fruit. It would be useful to have more flowering specimens for a more thorough comparison with *S. kaessneri*, which may reveal additional diagnostic characters. However, the two taxa are very clearly separated geographically by nearly 1,500 km and there are sufficient morphological differences for separation at the species level. A molecular phylogenetic study that includes the three taxa in this paper would also be useful to better understand relationships within *Synsepalum* and between *S. chimanimani*, *and S. kaessneri*. and *S. muelleri*. Whilst the molecular study by Borg et al. (2019) did not sample any of these species, it did include the type species of *Afrosersalisia*, *A. afzelii* (Engl.) A.Chev. (=*S. afzelii* (Engl.) T.D.Penn.) and one representative of *Vincentella*, *V. passargei* (Engl.) Aubrév. (= *S. passargei* (Engl.) T.D.Penn.), the segregate genus in which *S. muelleri* has previously been placed. They found that the clade containing *Afrosersalisia* is not closely related to the clades containing *Vincentella* or *Synsepalum* sensu stricto. This may support the morphological evidence that *S. chimanimani* and *S. muelleri* are not closely allied. However, it should be noted that *S. muelleri* (corolla lobes much shorter than tube) has a very different corolla morphology to *S. passargei* (corolla lobes much longer than tube; Kupicha 1983) and so the relationship between those species also requires confirmation.

Both *S. chimanimani* sp. nov. and *S. kaessneri* are rare species with restricted ranges and of conservation concern because of ongoing threats to the known populations. There are no known *ex situ* collections of either species (BGCI 2019) and it would be desirable to collect seeds for both banking and growing in botanic gardens.

### The botanical importance of the Chimanimani foothills: an Important Plant Area

The high Chimanimani Mountains have long been renowned for their high botanical endemism, particularly associated with the extensive outcrops of nutrient-deficient quartzites (Wild 1964; van Wyk and Smith 2001; Timberlake et al. 2016b; Wursten et al. 2017; Cheek et al. 2018). Much less well known is the botanical importance of the low elevation foothills of these mountains (c. 300–1200 m elevation, mainly below 1000 m), particularly to the south and east of the massif. This area has extensive stands of low altitude moist evergreen and semi-deciduous forest that, whilst now much fragmented, still represent the largest extent of this highly threatened habitat type in Mozambique.

The area of particular interest stretches from the lower valleys of the Rusitu and Haroni Rivers of Zimbabwe, the Makurupini area on the Zimbabwe-Mozambique border and the Maronga, Zomba and Mpunga communities of Mozambique. The botanical diversity of the Haroni-Rusitu-Makurupini region has been documented in an unpublished checklist of 787 species, compiled from a range of expeditions made between 1955 and 1998 (Timberlake 1999). Recent survey and inventory work in the Mozambican portion of this area, excluding Makurupini, documented 532 plant species (Timberlake et al. 2016a).

The forests vary in species composition and in the relative extent of evergreen versus deciduous components; the species composition in the Mozambican portion of these forests is discussed in detail by Timberlake et al. (2016a). Of particular interest botanically are the mostly evergreen forests and riverine fringes of Haroni-Makurupini, Maronga and the southwestern-most part of Zomba (Thekeza Forest). Amongst the dominant trees, these areas support what is potentially the largest population globally of *Maranthes goetzeniana* (Engl.) Prance. This species is often locally dominant in Maronga and also extends to Thekeza (Timberlake et al. 2016a).

Although of relatively low species richness compared to lowland forests in other parts of tropical Africa, the Chimanimani forests support a number of rare and threatened plant species. For example, the spectacular herb *Streptocarpus acicularis* I.Darbysh. & Massingue is so far known only from a single collection from along the Mevumozi River near Maronga (Darbyshire and Massingue 2014). *Vepris drummondii* Mendonça is largely restricted to the Haroni-Makurupini-Maronga forests except for an outlier population on nearby Mt Pene in Zimbabwe; it was encountered at low abundance in forests at Maronga in 2015 (I.D., pers. obs.). There is also a population of the globally Endangered wild coffee *Coffea salvatrix* Swynn. & Phillipson, or “mukofi” coffee, in the Maraumi Forest of Zomba and this species may occur more widely in this forest belt. Other range-restricted forest species include *Afrocanthium ngonii* (Bridson) Lantz and *Englerina swynnertonii* (Sprague) Polhill & Wiens.

These forests also support species that are nationally rare for Zimbabwe and/or Mozambique, including some interesting outlier populations. For example, *Ficus mucuso* Welw. ex Ficalho and *Raphidiocystis chrysocoma* (Schumach.) C.Jeffrey, West African species that are known in the Flora Zambesiaca region only from these forests, and *Dianella ensifolia* (L.) DC., a species of horticultural importance with an Indian Ocean distribution, on the African continent known in the wild only from the Chimanimani, Mabu and Ribáuè mountains of Mozambique. It is locally frequent at Maronga (Timberlake et al. 2016a). Also of interest is a dwarf, small-leaved species of *Podocarpus* (Podocarpaceae) that grows frequently along the rocky margins of forest rivers and streams at low elevations and also extending up into the higher mountains. This species has previously been placed within one of the two South African species *P. elongatus* (Aiton) L’Hér. ex Pers. (Farjon 2017) or *P. latifolius* (Thunb.) R.Br. ex Mirb. (Burrows et al. 2018) but it is quite possibly a distinct taxon – further taxonomic and molecular phylogenetic studies are required to confirm its placement. It is apparently also known from the Mafinga Mountains of Northeast Zambia which are also partially quartzitic (J. Timberlake, pers. comm.).

The forests are interspersed with extensive areas of miombo woodland, dominated by *Brachystegia* (most frequently *B. spiciformis* Benth.) and *Uapaca* species. Whilst important ecologically, the miombo does not contain high numbers of rare or threatened plant species. Of greater botanical significance is the presence of three other vegetation types within this mosaic:

1. Low elevation outcrops of nutrient-deficient quartzites, usually associated with light woodland dominated by *Brachystegia microphylla* Harms. These outcrops are most frequent in the Makurupini-Maronga area but also extend further east. They support an interesting rock flora including the endemic *Ficus muelleriana* C.C.Berg, a tiny fig that climbs on the rock faces, and *Otiophora lanceolata* Verdc., a locally abundant endemic shrublet. *Aloe ballii* Reynolds, including its two varieties var. ballii and var. makurupiniensis Ellert (both Vulnerable), is a delicate grass aloe restricted to quartzite slopes along the Zimbabwe-Mozambique border. Other range-restricted and/or scarce species of this habitat include *Sclerochiton coeruleus* (Lindau) S.Moore, *Gutenbergia westii* (Wild) Wild & G.V.Pope and *Sericanthe chimanimaniensis* Wursten & de Block ined. (see Burrows et al. 2018). Where quartzite outcrops along rivers and streams, the endemic grass *Danthoniopsis chimanimaniensis* (J.B.Phipps) Clayton can be locally frequent.

2. Seasonally wet grasslands, which occur in small scattered areas within the forest-woodland mosaic. These support an interesting, though not diverse, herb flora including the recently resurrected species *Crepidorhopalon flavus* (S.Moore) I.Darbysh. & Eb.Fisch. whose range is centred on the southern Chimanimani foothills (Darbyshire et al. 2019), and *Mesanthemum africanum* Moldenke, a Chimanimani endemic mainly found in the high mountains but which occurs at much lower abundance in these lowland wet grasslands (Timberlake et al. 2016a).

3. Swamps and lowland watercourses that are fringed by large stands of the striking tree *Pandanus livingstonianus* Rendle. Whilst fairly widespread, this tree has very isolated and localised populations and is thought to be threatened by habitat loss (Beentje 2009). The Zomba Centro Swamp is particularly important for this species.

The entirety of this area falls within the Chimanimani Trans-Frontier Conservation Area (TFCA), in part within the core TFCA, i.e. the Chimanimani National Park of Zimbabwe and Chimanimani National Reserve of Mozambique, and in part within the TFCA buffer zone. This buffer zone includes the Maronga, Zomba and Moribane Forest Reserves in Mozambique (see Müller et al. 2005). Lowland habitats within the core TFCA are largely intact, with only small areas of human encroachment at present. However, threats are severe within the buffer zone including within the Forest Reserves. Large areas of forest have either been cleared or degraded for subsistence agriculture, using fire to clear the undergrowth once the large trees have been felled. Excessive burning prevents forest regrowth and impacts other key habitats. Regular burning also encourages the continuing spread of the invasive South American shrub *Vernonanthura polyanthes* (Spreng.) A.J. Vega & Dematt. (syn. *V. phosphorica* (Vell.) H.Rob.; see Timberlake et al. 2016a; Sukhorukov et al. 2017) and this species is now mono-dominant over many hectares of disturbed, former forest habitats in the Chimanimani foothills, out-competing native species and preventing regeneration of natural habitats and encroaching into forest margins (Timberlake et al. 2016a). A further threat is the impact of mining for gold along some of the major rivers that flow from the massif, which pollutes the watercourses and denudes vegetation along their margins, as is clearly visible on satellite imagery on Google Earth. Conservation action is urgently needed in the TFCA buffer zone. Ongoing work with communities to attempt to better balance livelihoods with biodiversity conservation is ongoing, led by the Micaia Foundation (http://www.micaia.org), and this has led to the establishment of community conservation areas in the Maronga, Zomba and Mpunga communities of Mozambique (Timberlake et al. 2016a). However, there is still much work to be done to secure the future of the biodiversity within the TFCA buffer zone.

Applying the Important Plant Areas (IPA) criteria as revised by Darbyshire et al. (2017), the Chimanimani foothills qualify as an IPA under criterion A – presence of threatened species – with 17 criterion A taxa, of which 12 currently meet the threshold for criterion A(i) – presence of globally threatened species (see Table 2). This site will also qualify under IPA criteria B(ii) – exceptional number of species of high conservation importance, and C(iii) – presence of nationally threatened or range restricted habitat, due to the large extent of threatened lowland moist forest. The lowland forest and the high massif are combined together in the two Important Bird and Biodiversity Areas for Chimanimani, which are divided along the international boundary (BirdLife International 2019a, 2019b). However, in botanical terms, the species assemblages are markedly different between the Chimanimani foothills and the high massif (albeit with a mid-elevation transition zone) and the threats and conservation issues that the two areas face are also markedly different. With this in mind, we consider it most useful to recognise the Chimanimani foothills as an IPA in their own right, with the high massif recognised as a separate IPA. In doing so, this also helps to draw more attention to the importance of the Chimanimani foothills as a site of global importance for biodiversity and in urgent need of conservation action.

**Table 2.** Taxa that qualify the Chimanimani foothills as an Important Plant Area under criterion A: threatened species.

**Family**	**Taxon**	**IUCN Red List assessment**	**IPA sub-criteria met**	**Notes**
Asphodelaceae	*Aloe ballii* Reynolds	VU D2	A(i)	Two varieties, var. ballii and var. makurupiniensis
Asteraceae	*Gutenbergia westii* (Wild) Wild & G.V.Pope	VU B1ab(iii)+2ab(iii)	A(i)	
Commelinaceae	*Cyanotis chimanimaniensis* Faden ined.	Not Evaluated	A(iv)	Species currently under description
Fabaceae	Tephrosia longipes Meisn. var. swynnertonii (Baker f.) Brummitt	Not Evaluated	A(iv)	
Gesneriaceae	*Streptocarpus acicularis* I.Darbysh. & Massingue	CR B2ab(iii)	A(i)	
Linderniaceae	*Crepidorhopalon flavus* (S.Moore) I.Darbysh. & Eb.Fisch.	Not Evaluated	A(iv)	Provisionally assessed as Vulnerable – VU B1ab(iii) – by Darbyshire et al. (2019)
Loranthaceae	*Englerina swynnertonii* (Sprague) Polhill & Wiens	Not Evaluated	A(iii)	
Moraceae	*Ficus muelleriana* C.C.Berg	EN B1ab(iii)+2ab(iii)	A(i)	
Phyllanthaceae	Phyllanthus bernierianus Baill. ex Müll. Arg. var. glaber Radcl.-Sm.	Not Evaluated	A(iv)	
Poaceae	*Danthoniopsis chimanimaniensis* (J.B.Phipps) Clayton	EN B1ab(iii)+2ab(iii)	A(i)	
Rubiaceae	*Afrocanthium ngonii* (Bridson) Lantz	VU B1ab(iii)+2ab(iii)*	A(i)	
	*Coffea salvatrix* Swynn. & Phillipson	EN B2ab(i,ii,iii)	A(i)	
	*Otiophora lanceolata* Verdc.	VU B1ab(iii)+2ab(iii)	A(i)	
	*Sericanthe chimanimaniensis* Wursten & De Block ined.	VU B1ab(iii)+2ab(iii)*	A(i)	Species currently under description
Rutaceae	*Vepris drummondii* Mendonça	VU B1ab(iii)+2ab(iii)	A(i)	
Sapotaceae	*Synsepalum chimanimani* S.Rokni & I.Darbysh.	EN B1ab(iii)+2ab(iii)	A(i)	
Zamiaceae	*Encephalartos chimanimaniensis* R.A.Dyer & I.Verd.	EN B1ab(i,ii,iv,v)+ 2ab(i,ii,iv,v); C1	A(i) – see note	Requires confirmation that this species is still extant within proposed IPA

* publication pending

## Supplementary Material

XML Treatment for Synsepalum chimanimani

XML Treatment for Synsepalum kaessneri

XML Treatment for Synsepalum muelleri

## Supplementary materials

Supplementary material 1 Examined characters of *S. kaessneri* and the newly described species Data type: species data https://binary.pensoft.net/file/347393This dataset is made available under the Open Database License (http://opendatacommons.org/licenses/odbl/1.0/). The Open Database License (ODbL) is a license agreement intended to allow users to freely share, modify, and use this Dataset while maintaining this same freedom for others, provided that the original source and author(s) are credited.Saba Rokni, Bart Wursten, Iain Darbyshire
